# Supplementary material for: The effects of broadband elicitor duration on transient-evoked otoacoustic emissions and a psychoacoustic measure of gain reduction
Source: Res Sq. 2025 Aug 12:rs.3.rs-6753082. Originally published 2025 Jun 2. Preprint. [Version 2] doi: 10.21203/rs.3.rs-6753082/v2 (PMC12155214; doi:10.21203/rs.3.rs-6753082/v2)
Supplement: 1 [file NIHPPRS6753082V2-supplement-1.pdf]

Supplementary files

Supplementary File 1

Supplementary Figure 1. Schematic of gain reduction effects induced by the elicitor for each listening condition, illustrated using cochlear IO functions. Solid lines represent responses to the behavioral signal (denoted by bold S) or the TEOAE probe (denoted by bold P) within a cochlear filter centered at or near the signal/probe frequency. Responses with gain reduction are indicated by dashed lines positioned directly beneath the corresponding solid lines. A double-headed yellow arrow on the y-axis represents the threshold signal-to-masker ratio, which is assumed to remain constant across conditions. Psychoacoustic conditions are shown in panels A and B, which depict the off-frequency and on-frequency conditions, respectively, while panel C illustrates the no-masker condition. Panel D represents the TEOAE condition. The psychoacoustic signal and OAE probe are fixed on the lower linear portion of the IO function. The psychoacoustic conditions represent the horizontal displacement on the IO function while the OAE condition represents the vertical displacement on the IO function. Absolute thresholds are indicated by gray dashed horizontal lines in each panel.

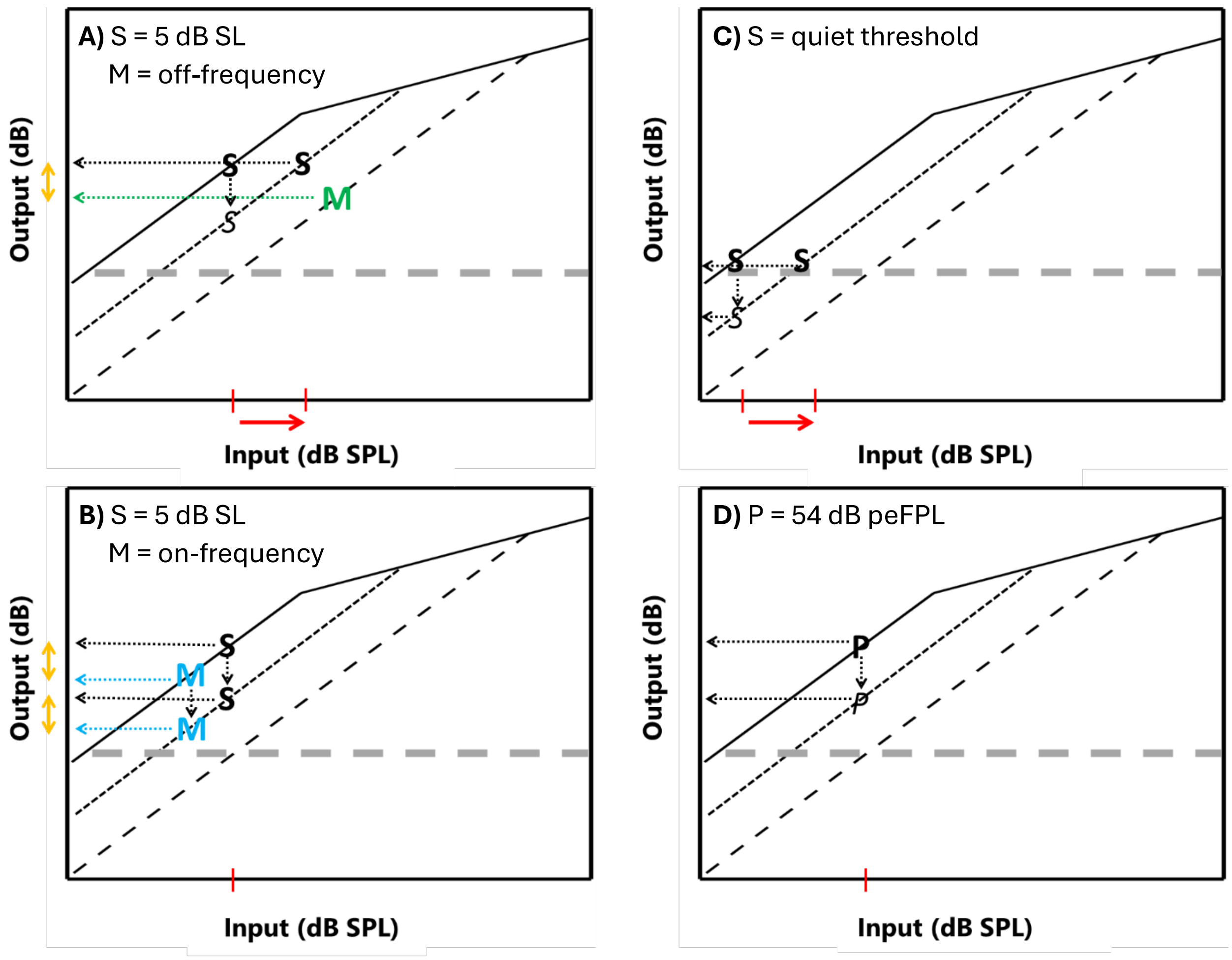
A) S = 5 dB SL
M = off-frequency
S
S
M
S
Output (dB)
Input (dB SPL)
C) S = quiet threshold
S
S
S
Output (dB)
Input (dB SPL)
B) S = 5 dB SL
M = on-frequency
S
M
S
M
Output (dB)
Input (dB SPL)
D) P = 54 dB peFPL
P
P
Output (dB)
Input (dB SPL)

Supplementary File 2

TABLE S1. Individual subjects' signal, masker, and masked thresholds for the conditions tested in the current study. Quiet thresholds served as the baseline threshold for the masker absent task and were used to determine the on- and off-frequency masker levels that shifted the signal by 5 dB. These on- and off-frequency masker levels were then fixed, and the signal thresholds for both on- and off-frequency conditions are reported here (i.e., the masker present baseline). Note that each subject's on- and off-frequency masked signal thresholds were always within 3 dB of one another, indicating equivalent masking of the signal. Each subject's gain estimate at the signal frequency was calculated by taking the difference in masked threshold between the off-frequency and on-frequency masked conditions when the signal was fixed at 5 dB SL.

Subjects	Quiet thresholds	On-frequency masker thresholds	Off-frequency masker thresholds	On-frequency signal thresholds	Off-frequency signal thresholds	Gain
S1	26.5	27.0	71.0	29.5	30.4	44.0
S2	23.5	24.0	74.0	30.6	30.8	50.0
S3	23.6	21.0	68.0	31.0	31.8	47.0
S4	24.5	25.6	67.0	30.6	30.8	41.4
S5	23.3	22.0	63.0	28.9	29.6	41.0
S6	29.5	26.0	64.0	36.9	35.6	38.0
S7	29.5	25.0	71.0	35.0	35.6	46.0
S8	20.9	18.2	60.2	26.0	24.8	42.0
S9	22.9	19.7	57.0	27.6	28.6	37.3
S10	20.6	17.3	72.0	24.9	23.8	54.7
S11	22.4	19.3	64.4	28.8	28.1	45.1
S12	26.3	24.4	64.5	32.1	29.1	40.1
S13	25.9	24.5	62.4	29.8	30.0	37.9
S14	26.6	23.3	66.2	29.8	29.8	42.9
S15	26.8	21.6	62.8	32.8	33.6	41.2
S16	20.8	17.8	57.0	26.5	25.8	39.2
S17	27.9	27.6	59.4	31.6	32.0	31.8
S18	27.6	22.8	57.9	29.0	29.3	35.1
S19	30.5	28.4	55.0	35.0	36.8	26.6
Average	25.2	22.9	64.0	30.3	30.3	41.1

Supplementary File 3

Supplementary Figure 2. TEOAE-induced phase shifts ($\Delta TEOAE_p$) as a function of elicitor duration. Individual subject data are shown as open circles connected by thin colored lines, while the average across subjects is shown by a thick purple line. Error bars on the averaged data reflect the standard error of the mean (SEM). Elicitor duration is plotted on the x-axis, and ΔTEOAEp (in degrees) is plotted on the y-axis. A negative phase shift indicates a phase lag, while a positive shift indicates a phase lead. A linear mixed-effects model (LMM) was used to examine whether elicitor duration predicted changes in $\Delta TEOAE_p$, the elicitor-induced phase shift in the TEOAE waveform. The model was specified as $\Delta TEOAE_p$ ~ Elicitor Duration + (1 | Subject), with $\Delta TEOAE_p$ as the dependent variable and *Elicitor Duration* entered as a categorical fixed effect with four levels (50, 100, 200, and 400 ms). A random intercept was included to account for subject-level variability. Prior to model fitting, two subjects (S5 and S7) were removed using a "leave-one-out" and "leave-two-out" strategy based on their influence on residuals and violation of interquartile range criteria across conditions. The final analysis included 17 subjects (n = 17). An ANOVA on the fixed effects (Type II Wald F-tests with Kenward-Roger degrees of freedom) revealed that elicitor duration failed to reach statistical significance, $F(3, 48) = 2.41$, $p = 0.078$. However, the variance associated with the random effect of subject ($\sigma^2 = 2.76$) exceeded the residual variance ($\sigma^2 = 2.44$), indicating substantial individual variability beyond within-subject error. See sections in the paper for interquartile range inclusion (Experiment 1) and LMM statistical parameterization (Comparisons of physiological and psychoacoustic measures of gain reduction). Although the fixed effect of elicitor duration was not statistically significant, the averaged $\Delta TEOAE_p$ data showed a small, monotonic trend toward increasing phase lead with longer elicitor durations, ranging from approximately ~0.7° at 50 ms to ~2.3° at 400 ms. This group-level trend was relatively subtle and remained consistent with and without the excluded outliers. However, individual responses varied considerably, with some subjects exhibiting little or no change in phase, while others showed clear phase lags or leads.

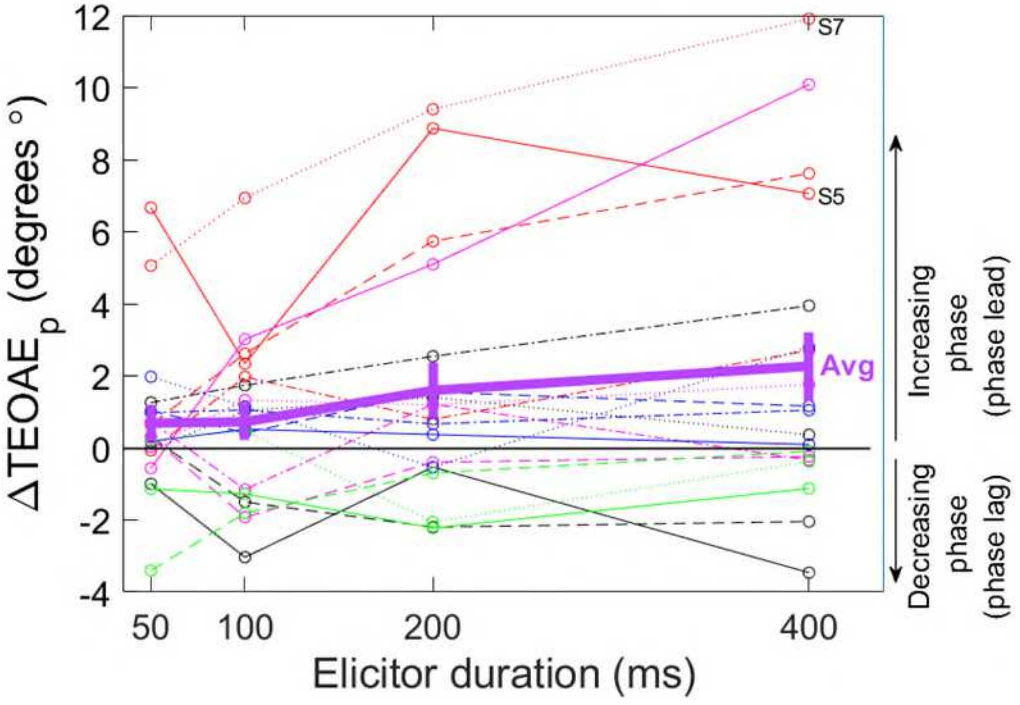

Supplementary Files

This is a list of supplementary files associated with this preprint. Click to download.

- SupplementaryFile1SupplementaryFigure1.png
- SupplementaryFile2SupplementaryTable1.pdf
- SupplementaryFile3SupplementaryFigure2.pdf
